# Monitoring of Geographic Atrophy Progression in Age-Related Macular Degeneration Using Fluorescence Lifetime Imaging Ophthalmoscopy

**DOI:** 10.1167/iovs.67.6.31

**Published:** 2026-06-16

**Authors:** Nicola Sagurski, Damian Jaggi, Joel-Benjamin Lincke, Sebastian Wolf, Martin S. Zinkernagel, Chantal Dysli

**Affiliations:** 1 Department of Ophthalmology, Inselspital, Bern University Hospital, University of Bern, Bern, Switzerland

**Keywords:** fluorescence lifetimes (FLTs), fundus autofluorescence (FAF), retinal imaging, fluorescence lifetime imaging ophthalmoscopy (FLIO), age-related macular degeneration (AMD), geographic atrophy (GA), disease progression

## Abstract

**Purpose:**

The purpose of this study was to examine fluorescence lifetime (FLT) changes within the border zone of geographic atrophy (GA) due to age-related macular degeneration (AMD) using fluorescence lifetime imaging ophthalmoscopy (FLIO).

**Methods:**

Patients with GA due to AMD were included. Fundus autofluorescence (FAF) intensity, spectral-domain optical coherence tomography (OCT), color fundus photography, and FLIO imaging were carried out at baseline and approximately 5 years of follow-up. GA progression was measured based on FAF images. FLT were measured at five different regions of interest: fovea, retina unaffected by GA, GA, and two areas directly next to the border zone of atrophy.

**Results:**

Thirty-two eyes of 17 patients (9 female patients, mean age = 77.4 years) were included. The GA area increased by an average of 1.21 mm^2^/year. At the GA border, FLT gradually decreased toward the unaffected retina at each visit and gradually increased in all zones over time. The FLT gradient at the border zone correlated significantly with the GA growth rate (*P* = 0.02, *r*^2^ = 0.17).

**Conclusions:**

Areas of GA showed significantly prolonged FLT compared to unaffected surrounding retinal tissue, confirming previous reports. The border zone FLT gradient was predictive of GA progression rate.

Age-related macular degeneration (AMD) is the leading cause of irreversible vision loss in the older population in the industrialized world.^1^^–^^3^ Globally, any stage of AMD was projected to affect approximately 196 million people in 2020 and 288 million by 2040, with AMD-related vision impairment (moderate to severe vision loss or blindness) affecting >8 million people worldwide in 2021.^4^^,^^5^ Geographic atrophy (GA) represents the end stage of non-exudative AMD. It is characterized by progressive atrophy of the retinal pigment epithelium (RPE) and irreversible loss of the overlying photoreceptors and outer retinal layers, as well as the underlying choriocapillaris. The often sharply demarcated geographical forms of atrophy in fundoscopy led to the name of GA.^6^ The exact pathomechanisms leading to advanced AMD with GA are not completely understood. Current evidence suggests that multiple processes contribute, including complement dysregulation, chronic inflammation, and mitochondrial stress in the RPE.^7^^–^^10^ In addition, genetic variants, such as Complement Factor H (CFH) or Age-Related Maculopathy Susceptibility 2 (ARMS2)/Human High-Temperature Requirement A-1 (HTRA1) have been implicated in increased risk of GA development.^11^^,^^12^ Further major risk factors are age, smoking, and nutrition. With respect to therapy, 2 intravitreal complement inhibitors targeting C3 and C5 have been approved in the United States since 2023 to slow down lesion enlargement. Although both agents demonstrated a moderate reduction in GA growth rates, no consistent improvement in visual acuity was observed in pivotal trials.^13^ ^14^ In Europe, neither treatment has received regulatory approval to date. Even with these therapeutic options, GA lesions continue to slowly but steadily enlarge over time.^15^ In some cases, the central macula is spared (foveal sparing [FS]) leaving a central patch of preserved RPE and photoreceptors, through which a certain degree of visual acuity can be retained for a time. For longitudinal monitoring of initial extent and disease progression, fundus autofluorescence (FAF) intensity has been the imaging modality of choice, which is capable of delineating the outlines of GA.^16^^,^^17^ With its help, various different growth patterns of GA have been described.^18^ Another imaging modality, which can depict GA, is fluorescence lifetime imaging ophthalmoscopy (FLIO). It is a relatively novel imaging technique, which measures the fluorescence lifetimes (FLTs) of retinal fluorophores, providing insight into retinal metabolic processes.^19^^–^^22^ Whereas autofluorescence intensity reflects the presence (bright) or absence (dark) of retinal fluorophores, particularly those originating from the RPE, FLT imaging adds an additional biomarker dimension. Weak fluorophores that are barely or not detectable in FAF intensity measurements may nonetheless exhibit distinct FLTs. Differentiation of FLTs within GA and its border zones may therefore provide further insights into the spatial distribution of retinal fluorophores and associated metabolites. Dysli et al. previously analyzed a cohort of patients with GA using FLIO.^23^ Sauer et al. described FLT patterns in patients with GA and FS^24^ and we have published longitudinal data of patients with FS.^25^ These FLIO measurements showed significantly prolonged FLTs in areas affected by GA, indicating a loss of retinal fluorophores with short FLTs.

The goal of this study was to provide additional longitudinal data regarding changes in GA and its border zones, its progression speed, and associated visual acuity changes, and to correlate these findings with changes in the FLIO measurements.

## Methods

The study is registered at ClinicalTrials.gov (NCT01981148). It was approved by the local ethics committee, and the procedures followed the tenets of the Declaration of Helsinki and the International Ethical Guidelines for Biomedical Research involving Human Subjects (Council for International Organizations of Medical Sciences). Written informed consent was obtained from all patients before study enrollment.

### Patients and Examinations

This prospective cohort study included eyes from patients with GA secondary to non-exudative AMD, recruited from the outpatient Department of Ophthalmology at the University Hospital of Bern, Switzerland. Exclusion criteria were signs or history of neovascular AMD, significant cataract, and other ocular pathologies, which could significantly affect measurements, such as media opacities or non-AMD retinal disease. To further exclude the influence of the crystalline lens, the pseudophakic subgroup was separately analyzed. GA was defined as a hypofluorescent lesion in FAF imaging with clearly demarcated borders, a minimum size of 1.27 mm^2^ (½ disc area), and was required to meet the optical coherence tomography (OCT)-based criteria for atrophy classification as defined by spectral-domain OCT imaging.^26^ The examination consisted of slit-lamp biomicroscopy with dilated fundoscopy and assessment of the best corrected visual acuity (BCVA). The multimodal imaging included FAF intensity, color fundus photography, OCT, and FLIO images. Patients were seen at 2 to 3 visits over the duration of 4 to 7 years. Follow-up visits were aligned with routine clinical consultations rather than fixed intervals.

### FLIO Measurement

**Figure 1.**
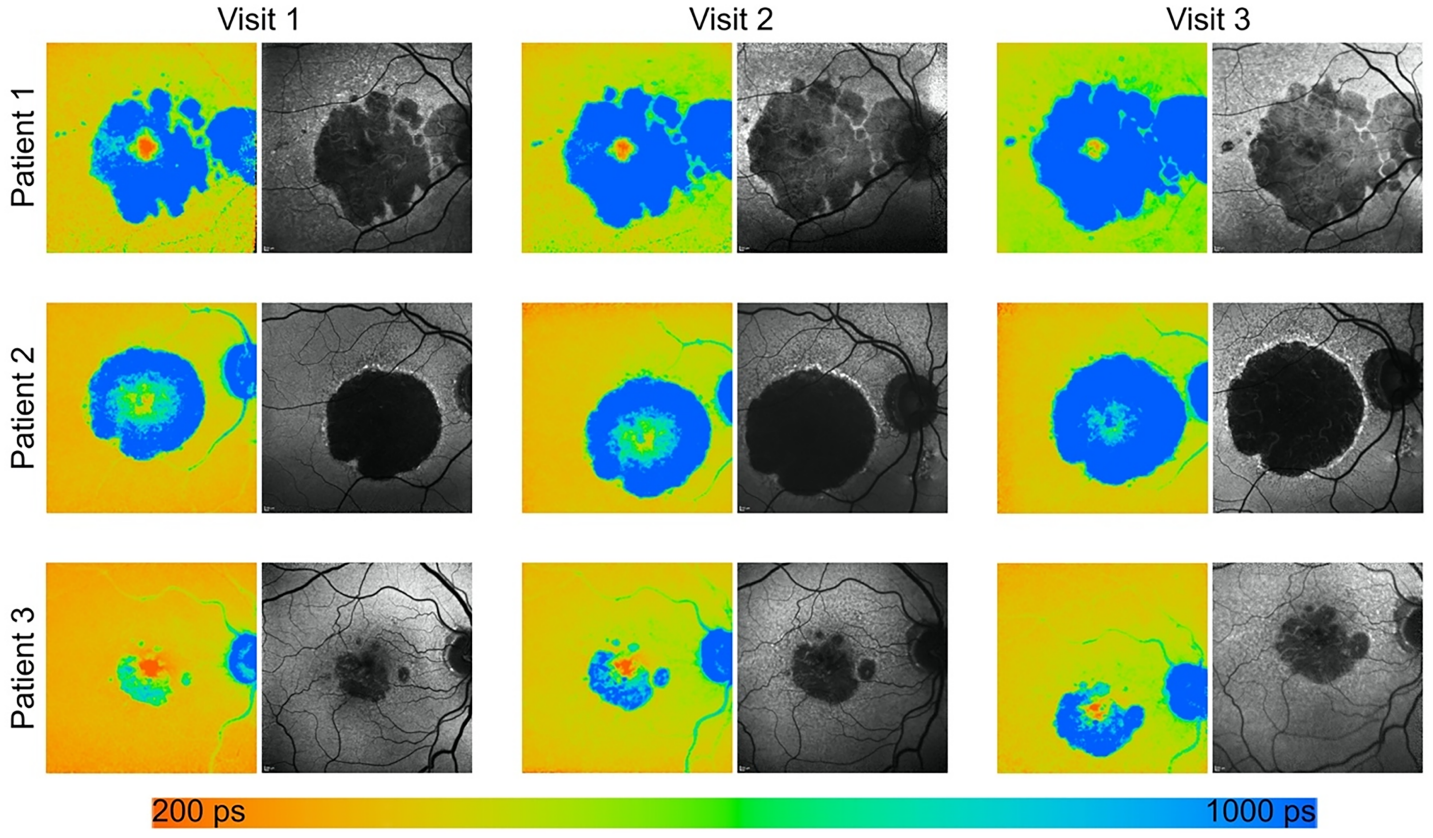
Representative fluorescence lifetime imaging ophthalmoscopy (FLIO) and fundus autofluorescence (FAF) images of three patients with geographic atrophy (GA). Growth of GA is demonstrated over up to three visits with the aforementioned imaging modalities.

FLTs of the retina were acquired using an FLIO device from Heidelberg Engineering (Heidelberg, Germany). Representative images are displayed in Figure 1.

The FLIO device radiates with a 473 nm pulsed blue laser at an 80-megahertz (MHz) repetition rate for excitation of retinal autofluorescence. The emitted fluorescence is registered by time-correlated single-photon counting (TCSPC) modules using two highly sensitive hybrid photon-counting detectors (Becker & Hickl, Berlin, Germany). Following detection channels with distinct wavelength ranges were used: a short spectral channel (SSC; 498–560 nm) and a long spectral channel (LSC; 560–720 nm). A confocal high-contrast infrared image was recorded simultaneously to ensure that each recorded photon is localized at the correct spatial location. A photon count of 1000 photons per pixel was acquired at least for every location within the image. FLIO image acquisition took approximately 1.5 to 3 minutes per eye.

### Analysis of Fluorescence Lifetime Data

The Becker & Hickl software (SPCImage version 4.4.2) was used for the analysis of recorded lifetime data. A biexponential decay model was applied using a binning factor of one. This procedure resulted in a short (T1) and a long (T2) lifetime component with corresponding amplitudes a1 and a2.

**Figure 2.**
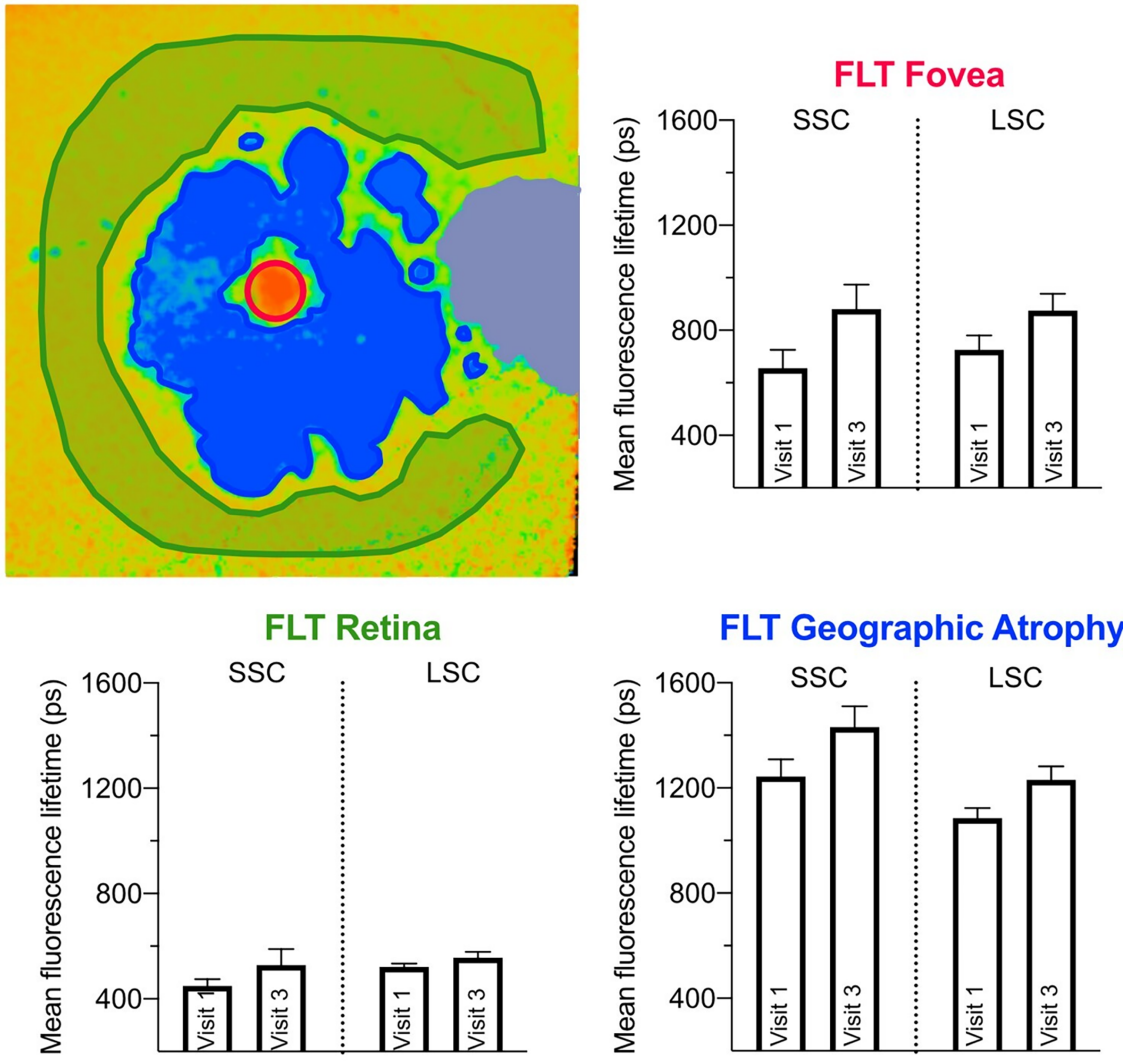
Representative FLIO image of foveal sparing GA showing the individual zones, in which lifetimes were measured and averaged. Peripapillary atrophy was excluded (*grey area*). Fluorescence lifetimes (FLTs) where measured in the foveal (*red*), geographic atrophy (*blue*), and retinal (*green*) areas in a short (SSC) and a long (LSC) spectral channel over up to three visits. The graphs depict the baseline (visit 1) and end-of-study (visit 3) FLT measurements. PS, picoseconds.

**Figure 3.**
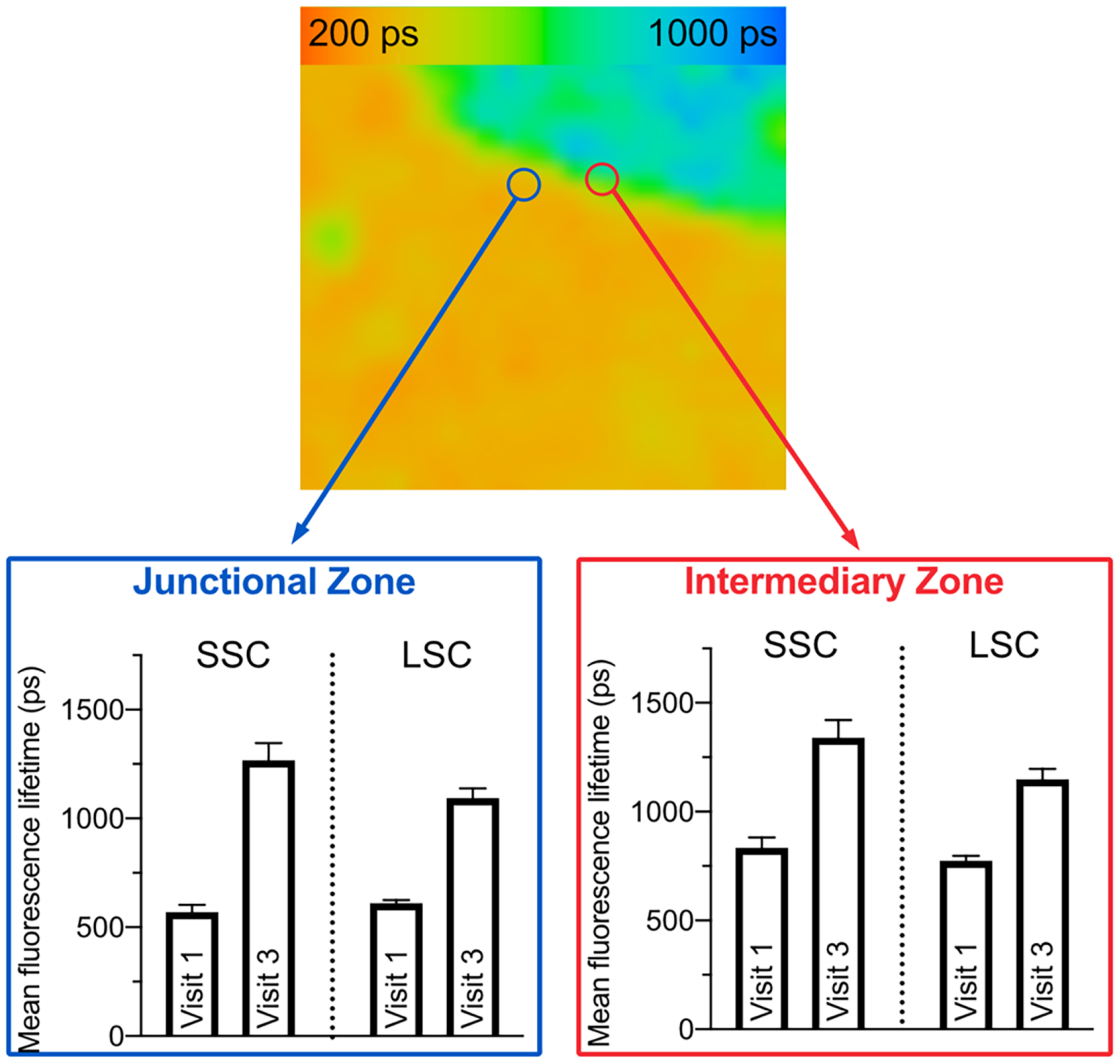
Representative FLIO image illustrating the position of the two regions of interest (ROI) defining the border region. In the graphs, the mean FLT for these regions is depicted for baseline and visit three in the short (SSC) and a long (LSC) spectral channel.

For topographical mapping and quantitative analysis of measured FLTs, the software “FLIO Reader” (ARTORG Center for Biomedical Engineering Research, University of Bern, Bern, Switzerland) was used. FLTs were measured in the following specific regions of interest (ROI), as depicted in Figure 2 and Figure 3:

1. Fovea: Defined by a circle (diameter of 300 µm) centered on the foveola.
2. Retina: Defined as healthy retina with a minimal distance of 500 µm to areas affected by GA.
3. GA: Defined as areas of complete RPE and outer retinal atrophy (cRORA, following the definition of GA consensus guidelines).^26^
4. Border area of GA, further subdivided into:
  1) Intermediary Zone (IZ): First band around GA, which exhibits slightly shorter FLT compared to GA. On FAF-imaging indistinguishable (same hypofluorescence as GA). We defined it as a band of 100 µm in width, beginning at the point of initial FLT decrease after the area of homogenously prolonged FLT with GA.
  2) Junctional Zone (JZ): Outermost rim of GA, directly adjoining the aforementioned IZ. On FAF imaging, it was often hyperautofluorescent. We defined it as the region sandwiched between the IZ and healthy retina with a width of 100 µm.

**Figure 4.**
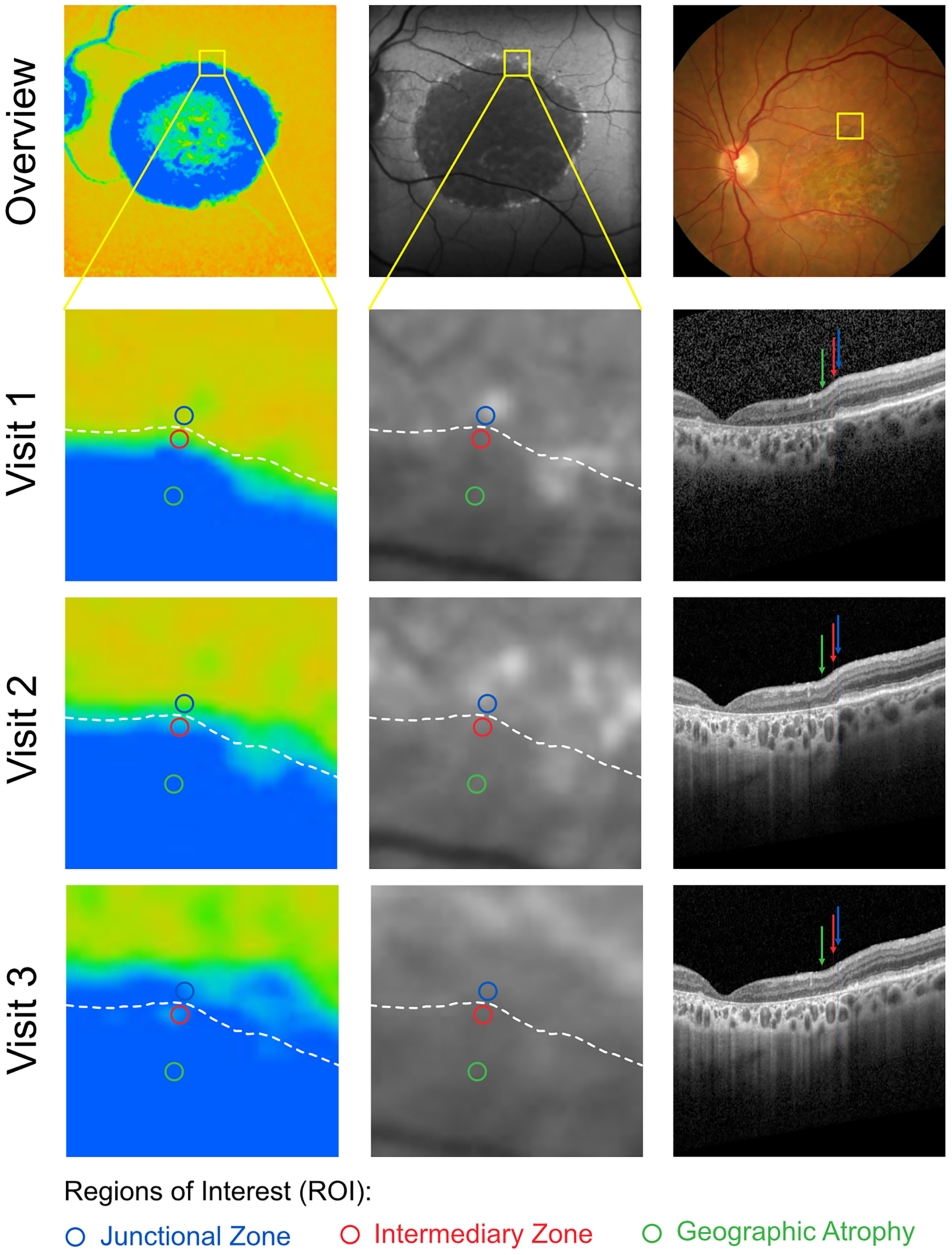
Borders of geographic atrophy were classified into two distinct regions of interest (ROI): intermediary zone (IZ; *red circle*) and junctional zone (JZ; *blue circle*). Additionally, the ROI geographic atrophy is marked (GA; *green circle*). Over three visits, fluorescence lifetimes (FLTs) were measured at these ROIs. When GA progressed it usually enclosed them. The *left row* displays the FLIO images, whereas the *middle row* displays the corresponding FAF, and on the *right row* the corresponding OCTs, marked with *arrows* at the position of the ROIs, can be seen. The *dashed white line* in the FLIO and FAF images represents the margin between clearly demarcated hypofluorescence and healthy retina in FAF images at baseline. It acts as reference to better illustrate the growth in the consequent visits. It should be noted, that the measuring positions for the individual ROIs do not change over time.

In each ROI, five manually selected representative circular measurements with a diameter of 100 µm for the fovea, IZ, and JZ, and of 200 µm for the retina and GA, were placed. The foveola was identified on the simultaneously recorded infrared image and verified on OCT. Unaffected retina was sampled by placing 5 circular ROIs at least 500 µm from the GA border while avoiding vessels and obvious imaging artifacts and confirming absence of atrophy on OCT. The 100 µm band width (IZ/JZ) and ROI sizes were chosen to standardize measurements across eyes and visits. Mean FLT (*Tm*) as well as the individual components *T1* and *T2* and amplitudes a1 and a2 were recorded for each ROI. *Tm* represents an amplitude-weighted average of the decay parameters *T1* and *T2*. The five individual measurements of each ROI were subsequently averaged for further analysis. During follow-up, ROI locations were kept consistent across time points using retinal landmarks; however, zone assignment could shift as GA enlarged over time (Fig. 4).

In addition to absolute FLT values, we quantified the border zone FLT gradient at baseline as the difference between the intermediary zone and junctional zone (ΔFLT_IZ – JZ = FLT_IZ − FLT_JZ). This parameter was correlated with the square-root–transformed GA growth rate for both channels.

### Calculation of GA Growth Speed

FAF images were acquired during FLIO image acquisition using the inbuilt 473 nm laser as the excitation source. For GA area calculation, the open-source software ImageJ 1.53a (National Institutes of Health, Bethesda, MD, USA) was used. Images were analyzed using a semi-automated threshold processing to quantify the GA-affected area. The included area was verified for the aforementioned GA criteria using OCT. Structures with similar grey levels, such as vessels as well as peripapillary atrophy, were excluded. The measured area of atrophy was subsequently square root transformed to decrease the association with the lesion size.^27^

### Statistical Analysis

For statistical analysis, Prism 9 software (GraphPad Software Inc., La Jolla, CA, USA) was used. Data were analyzed for normality using the d’Agostino-Pearson omnibus K2 test. Descriptive statistics were used for demographic, baseline, and general imaging values. Pearson correlation with Benjamini-Hochberg false discovery rate (FDR) adjusted p values were used for the correlation of border zone contrast and growth rate. *P* values of ≤ 0.05 were considered as statistically significant.

## Results

Thirty-two eyes of 17 patients (9 female patients) were analyzed. The mean age of patients at baseline (visit 1) was 78.1 years (range = 70–90 years) and 83.2 years (range = 75–95 years) at the last visit (visit 3). The mean duration between baseline and last visit was 5.1 years (SD ± 0.85 years, range = 4.5–7.1 years). A subgroup of 16 eyes (8 patients) was examined at an interim visit (Table 1).

**Table 1.** Patient and Ocular Characteristics

	Visit 1 (*n* = 32 Eyes)	Visit 2 (*n* = 18 Eyes)	Visit 3 (*n* = 32 Eyes)
Timeline, mo	0	31.9	61.9
Age, y	77.4 (66 – 90)	77.8 (68 – 86)	82.44 (70 – 95)
Gender	16 F/16 M	10 F/8 M	16 F/16 M
GA lesion size, mm^2^	11.4 ± 1.4	18.7 ± 2.1	17.48 ± 8.7
BCVA, letters	42 ± 4	38 ± 8	33 ± 4
FLT fovea, SSC/LSC, ps	646 ± 74/720 ± 58	848 ± 149/850 ± 104	866 ± 99/871 ± 68
FLT geographic atrophy, SSC/LSC, ps	1233 ± 68/1084 ± 41	1570 ± 99/1293 ± 63	1428 ± 84/1235 ± 54
FLT retina, SSC/LSC, ps	452 ± 27/527 ± 13	574 ± 57/596 ± 24	536 ± 64/561 ± 22
FLT intermediary zone, SSC/LSC, ps	823 ± 53/770 ± 27	1394 ± 112/1134 ± 60	1332 ± 90/1151 ± 54
FLT junctional zone, SSC/LSC, ps	564 ± 38/608 ± 18	1223 ± 108/1042 ± 65	1254 ± 89/1090 ± 52

### Geographic Atrophy Area and Progression

**Figure 5.**
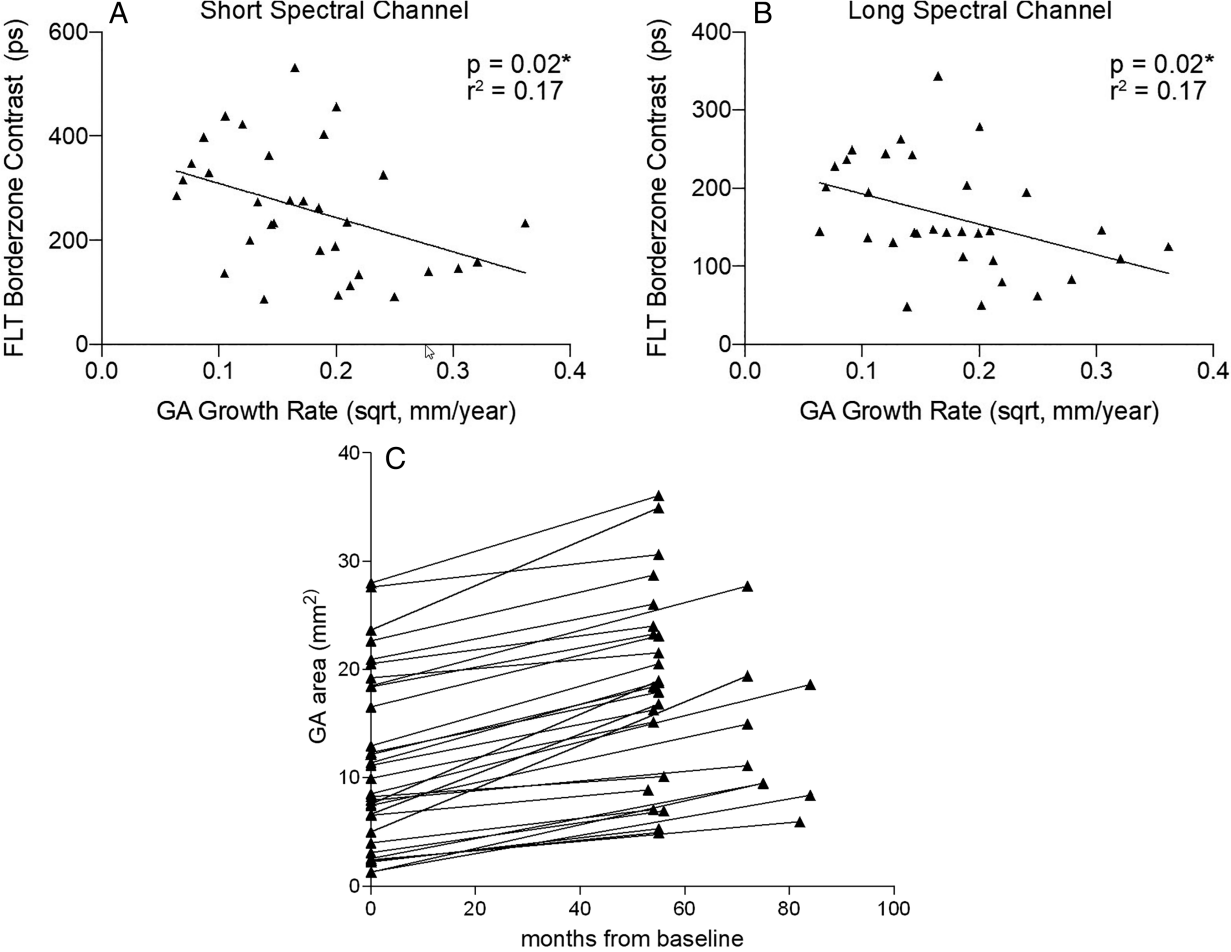
(**A,** **B**) Correlation between the fluorescence lifetime (FLT) contrast of the border zones (at the baseline visit) and the average GA progression rate of the following years for the short **A** and the long **B** spectral channel. The border zone contrast is defined by the difference in FLT between the intermediary zone and the junctional zone. The GA growth rate is square root transformed (sqrt) to reduce the influence of the individual lesion size. (**C**) Shows the GA growth over the whole study period for each patient.

Mean retinal area affected by GA (± standard error of the mean “SEM”) was 11.35 ± 1.4 mm^2^ or (sqrt) 3.14 ± 0.22 mm at visit 1 and 17.48 ± 1.53 mm^2^ or (sqrt) 4.05 ± 0.19 mm at visit 3. BCVA decreased by (mean ± SEM) 9.8 ± 2 letters from visit 1 to visit 3. The mean increase of GA-affected area was 1.21 ± 0.1 mm^2^ per year or (sqrt) 0.18 ± 0.01 mm per year, as illustrated as a line chart in Figure 5.

### Dynamics of Fluorescence Lifetimes

FLT increased in all measured ROIs with time, as shown in Figure 2 and Figure 3. The most pronounced prolongation in FLT between visit 1 and 3 was seen in the JZ with a mean prolongation (±SEM) of 690 ± 97 picoseconds (ps) in the SSC and 482 ± 55 ps in the LSC. The smallest change was seen in areas of unaffected retina with a mean prolongation of 81 ps and 34 ps in the SSC and LSC, respectively. All FLT measurements for all ROIs at each visit are listed in Table 1. Further dynamics of the FLT including multimodal imaging are illustrated in Figure 4.

### Correlation With Progression Rate

At baseline, the border zone FLT contrast (ΔFLT_IZ–JZ) correlated significantly and inversely with the square-root–transformed GA growth rate (*P* = 0.021, *r*² = 0.17; SSC and LSC; see Fig. 5), with lower IZ–JZ contrast being associated with faster GA progression. The relationship is also observed at the interim visit with the following correlation parameters: SSC: *r* = −0.57, FDR = 0.042, LSC: *r* = −0.50, FDR = 0.051.

### Pseudophakic Group

To exclude the influence of cataract progression on FLT measurements, a purely pseudophakic subgroup analysis with 22 eyes was formed. As expected, the average FLT was shorter than in the mixed group due to the removed influence of the crystalline lens.^28^ We globally observed similar results in this subgroup as in the complete cohort. In addition, the correlation between the square root transformed growth per year and IZ–JZ FLT difference at visit 1 remained statistically significant (FDR adjusted *P* value for the pseudophakic subgroup; *P* = 0.024 for both SSC and LSC). Table 2 shows an overview of the results of the pseudophakic subgroup.

**Table 2.** Pseudophakic Subgroup

	Visit 1 (*n* = 22 Eyes)	Visit 2 (*n* = 10 Eyes)	Visit 3 (*n* = 22 Eyes)
Timeline, mo	0	25.2	58.8
Age, y	79.4 (71 – 90)	79.8 (76 – 84)	84.3 (75 – 95)
GA lesion size, mm^2^	11.70 ± 7.9	19.21 ± 8.7	17.71 ± 9.1
BCVA, letters	44 ± 5	39 ± 10	35 ± 6
FLT fovea, SSC/LSC, ps	504 ± 74/613 ± 60	494 ± 113/629 ± 84	654 ± 78/736 ± 65
FLT geographic atrophy, SSC/LSC, ps	1068 ± 61/994 ± 43	1298 ± 61/1130 ± 49	1263 ± 51/1132 ± 36
FLT retina, SSC/LSC, ps	376 ± 21/494 ± 13	406 ± 11/533 ± 13	414 ± 7/516 ± 12
FLT intermediary zone, SSC/LSC, ps	688 ± 43/708 ± 26	1072 ± 46/965 ± 27	1174 ± 51/1059 ± 36
FLT junctional zone, SSC/LSC, ps	453 ± 28/556 ± 16	924 ± 66/874 ± 39	1088 ± 50/993 ± 34

## Discussion

Measurements of FLT with FLIO in areas affected by GA were reported to be significantly longer compared with healthy, unaffected areas.^23^^,^^24^ Consistent with these findings, our data showed markedly longer FLT within GA lesions compared to healthy retina in both spectral channels. In the border zone of GA, a gradual decrease of FLTs toward the healthy unaffected retina was observed.^29^^,^^30^ This gradient can be easily recognized in the color-coded FLIO maps as a rim of greenish color encompassing areas of GA. This region has been described as “adjacent zone” or “junctional zone” in previous publications concerning FLIO and GA.^23^^,^^24^ This hyperfluorescent border zone can exhibit variable expression and patterns, which were the target of previous studies, where a correlation between the growth rate of GA and FAF patterns were detected.^31^^,^^32^ Patterns with more abundance of hyperfluorescence and diffuse spread were associated with significantly faster growth rates compared to more muted continuous or focal patterns.^32^ Furthermore, a positive correlation between the total area of hyperfluorescence and the growth rate was found.^33^ In histologic examinations, this hyperfluorescence at the edge of GA was associated with vertical superimposition of RPE cells, providing a possible explanation for the increase of signal intensity in FAF imaging.^34^ With FLIO, a gradual decrease in FLT toward healthy retina beginning in the outermost part of the GA lesion was measured.

The mean GA growth rate in our cohort was 1.21 ± 0.1 mm^2^ per year or (sqrt) 0.18 ± 0.01 mm per year, which is comparable with previously published results albeit at the lower end of reported ranges.^35^^,^^36^ Growth rate was described to be dependent on the lesion size, which might explain this result.^37^ To prevent confounding with the lesion size, we used the square root transformations as previously described.^27^ There are various proposals for predicting the growth rate in GA. The reliable prediction of GA progression rate is of great interest also in view of novel therapeutic possibilities for GA. As mentioned before, the patterns of hyperfluorescence depicted on FAF can give an indication of the expected growth rate. Recent developments have increasingly focused on artificial intelligence-based prediction of GA progression.^38^ Deep learning models trained on multimodal imaging, including FAF and OCT, have demonstrated the ability to estimate individual lesion growth rates with moderate to high accuracy.^39^^–^^41^ Moreover, it has been shown that even single-modality FAF input can be sufficient to forecast short-term enlargement patterns using convolutional networks.^2^ In more recent comparisons, artificial intelligence (AI)-based prediction models even outperformed retinal specialists in estimating GA progression speed.^3^ To complement these AI-based pattern prediction strategies, we investigated whether functional lifetime characteristics could serve as biological predictors of progression. In search of these markers, we correlated the FLT parameters and contrasts between the individual measured ROIs with the corresponding growth rates. We found a significant negative association between GA growth rate and the border zone FLT contrast (IZ–JZ) at baseline: rapidly progressing GA was associated with a lower IZ–JZ FLT contrast. This likely reflects a more homogeneous FLT profile across the GA margin at baseline, driven by relative changes in the IZ, the JZ, or both.

A limitation of this study is the measurement accuracy of longitudinal data with FLIO. As there is no inbuilt follow-up mode in the software, recurring measurements at exactly the same spot turned out to be challenging. We chose easily recognizable landmarks for these spots in the FAF image, such as specific vessel configurations, as the reference point but in cases of rapidly expanding GA, even these landmarks can become partly obscured. However, for the FLT measurements at the baseline visit, on which the correlation predicting growth is based upon, this circumstance had no influence. Another limitation is the inclusion of phakic patients, although the majority was pseudophakic (22 of 32 eyes). To minimize the possibility that the observations are based on increasing lifetimes due to cataract progression, we analyzed a purely pseudophakic subgroup. In this subgroup, we were able to reproduce the same results as in the complete cohort. Another limitation is the high variability in the follow-up windows due to rescheduled clinical visits and the coronavirus disease (COVID) pandemic. On the technical level, the study is furthermore limited by the fact that it is not yet possible to calculate specific fluorophores out of the FLIO signal, and therefore interpretation of the FLIO signal is challenging.

## Conclusions

Our results confirm findings from previous studies, where prolonged FLT have been described in GA. Longitudinal analysis shows further continuous FLT prolongation, presumably due to the ongoing loss of retinal fluorophores. The FLT gradient at the border zone of the GA indicates progression rate for the following years. Therefore, FLIO might have the potential to serve as a monitoring tool for therapeutic interventions as it might be capable of detecting early retinal molecular changes associated with therapy responses in border zones of GA.
